# Propagation of Tau via Extracellular Vesicles

**DOI:** 10.3389/fnins.2019.00698

**Published:** 2019-07-02

**Authors:** Mar Pérez, Jesús Avila, Félix Hernández

**Affiliations:** ^1^Departamento de Anatomía Histología y Neurociencia, Facultad de Medicina UAM, Madrid, Spain; ^2^Network Center for Biomedical Research in Neurodegenerative Diseases (CIBERNED), Madrid, Spain; ^3^Centro de Biología Molecular Severo Ochoa (CSIC-UAM), Madrid, Spain

**Keywords:** tau propagation, extracellular vesicle, neurodegenerative disease, tau protein, Alzheimer’s disease

## Abstract

Extracellular vesicles (EVs), like exosomes, play a critical role in physiological processes, including synaptic transmission and nerve regeneration. However, exosomes in particular can also contribute to the development of neurodegenerative conditions such as Alzheimer’s disease (AD), Parkinson’s disease, and prion diseases. All of these disorders are characterized by protein aggregation and deposition in specific regions of the brain. Several lines of evidence indicate that protein in exosomes is released from affected neurons and propagated along neuroanatomically connected regions of the brain, thus spreading the neurodegenerative disease. Also, different cell types contribute to the progression of tauopathy, such as microglia. Several groups have reported tau release via exosomes by cultured neurons or cells overexpressing human tau. Although the exact mechanisms underlying the propagation of protein aggregates are not fully understood, recent findings have implicated EVs in this process. The AD brain has two hallmarks, namely the presence of amyloid-β-containing plaques and neurofibrillary tangles, the latter formed by hyperphosphorylated tau protein. Both amyloid peptide and tau protein are present in specific exosomes. This review summarizes recent advances in our understanding of exosomes in the pathology of AD, with a special focus on tau protein.

## Introduction

Brain microtubules were first isolated and characterized in the 1970s (Weisenberg, 1972). This study revealed the presence of a main protein, tubulin, and several others described as microtubule-associated proteins (MAPs) in these structures. One of these MAPs, tau, was first characterized by Kirschner’s group (Weingarten et al., 1975). Similar to other MAPs, the function of tau was found to be related to an increase in microtubule stabilization (Drubin and Kirschner, 1986), which prevents cell proliferation and facilitates neuronal differentiation. Recent discoveries about partners (End binding proteins 1 and 3, tRNA), different subcellular localizations (nucleus, nucleolus, plasma membrane, dendrites and dendritic spines) or association with cellular organelles (ribosomes, endoplasmic reticulum and the Golgi apparatus) for tau suggest additional roles. According to these studies, tau should be implicated on mechanisms of synaptic plasticity, structural architecture of heterochromatin, chromosome stability or regulating the cellular transcriptome (for more detail see review Sotiropoulos et al., 2017).

Although tau is mainly an intracellular protein, there are reports indicating that extracellular tau is present in brain interstitial fluid (Yamada et al., 2011), its amount decreasing in this medium during sleep (Lucey et al., 2019). Secreted tau may be implicated in some features of sleep (Cantero et al., 2010; Lucey et al., 2019). However, independently of this notion, secreted tau protein is present *in vivo*, and its secretion appears to be regulated.

## Tau Secretion and Its Regulation

The presence of extracellular tau suggests that it is secreted under physiological conditions. In this regard, it has been proposed that intracellular tau is released upon an increase in neuronal activity (Pooler et al., 2013; Yamada et al., 2014). In addition to neuron activity-dependent tau secretion, the extracellular form of the protein may arise by other mechanisms, such as neuron death (Gomez-Ramos et al., 2006), intracellular tau accumulation (Simon et al., 2012), a tauopathy (Clavaguera et al., 2009), or modulation by tau mutations (Karch et al., 2012). In the case of neuron death, intracellular proteins like tau are released into the extracellular space.

Intracellular tau accumulation can arise due to aging. In this regard, tau accumulation in older adults is associated with hippocampal hyperactivity (Huijbers et al., 2019). Also, an increase in intracellular levels of tau can result from pathological disorders related to a decrease in the turnover of this protein. This reduction can be caused by impaired tau degradation through two main systems, the ubiquitin-proteasome pathway and the autophagy-lysosomal pathway (Wang et al., 2009; Chesser et al., 2013; Lee et al., 2013; Guo et al., 2017). Recently, a third degradative pathway, the endolysosomal system, has been proposed for neurodegenerative disorders such as AD or Parkinson’s disease (Vaz-Silva et al., 2018). Rab35 and the endosomal sorting complex required for transport (ESCRT) machinery should be involved in the delivery of tau to lysosomes via early endosomes and multivesicular bodies. Intracellular tau accumulation may facilitate post-translational modifications, like phosphorylation or truncation, in the protein (Avila et al., 2004), and the modified tau isoform can also be secreted.

Hyperphosphorylation is one of the most important post-translational modifications in AD and related tauopathies (Medina et al., 2016). An increase in phosphorylation at T181 (Vanmechelen et al., 2000) or T231 (Hampel et al., 2005) has been described in the cerebrospinal fluid of AD patients, although a decrease with the progression of AD has also been reported (Hampel et al., 2001). Thus, a tau mutant mimicking phosphorylation is more efficiently secreted than one mimicking dephosphorylation in Hela cells (Plouffe et al., 2012). However, it is still unclear whether phosphorylation regulates tau secretion, since both phosphorylated and unphosphorylated tau species have been detected in the extracellular space. Studies carried out in primary cortical neurons showed the release of unphosphorylated tau in control conditions (Pooler et al., 2013), while other groups have reported that cortical neurons secrete phosphorylated and unphosphorylated Tau species in response to various insults (Plouffe et al., 2012). Further research is needed to elucidate how tau phosphorylation contributes to the secretion of this protein. However, one point to keep in mind is the observation that extracellular tau is dephosphorylated in the AD brain by tissue Non-specific alkaline phosphatases (Diaz-Hernandez et al., 2010).

## Tau Secretion in Cell Culture Models

The transfer of tau in Non-neuronal cell cultures may take place upon tau secretion (Frost et al., 2009), and in a direct way it has been demonstrated that the accumulation of tau in Non-neuronal cells promotes its secretion to the extracellular space in nacked (free) form or via membrane vesicles (Simon et al., 2012). This secretion could occur by accumulation of the whole tau molecule or by accumulation of tau fragments (Perez et al., 2016). Post-translational modifications like phosphorylation or truncation may regulate tau secretion in Non-neuronal cell models (Diaz-Hernandez et al., 2010; Plouffe et al., 2012). Also, secretion of truncated tau forms has been reported in neuronal cells (Kim et al., 2010; Kanmert et al., 2015). In this regard, a number of mechanisms explaining the secretion of truncated (and/or aggregated) tau and tau in free form (Kfoury et al., 2012) have been put forward.

Regarding the factors involved in the secretion of tau in cell culture models, End-binding proteins bind to the N-terminal end of human tau protein (Sayas et al., 2019). This observation suggests that the interaction of EB proteins with tau facilitates the localization of tau close to cellular membrane and its further secretion. However, more analysis is needed to confirm this notion.

## Possible Mechanisms of Tau Secretion

The molecular mechanisms responsible for the secretion of tau in its different forms: unmodified, phosphorylated, truncated, etc, are unclear. It has been postulated that secretion takes place through a Non-vesicular (free protein) secretory pathway, because tau lacks a signal sequence to regulate its transport to the endoplasmic reticulum, a step needed in the conventional secretary pathway (Yamada, 2017). On the other hand, the Golgi dynamics in neurons has been linked to the regulation of tau secretion (Mohamed et al., 2017). Also, mitochondria damage in neurons and Non-neuronal cells may also be involved in the modulation of tau secretion (Shafiei et al., 2017). Although it is not clear how tau can be localized at the cell membrane, several reports demonstrate its presence at the membrane (Brandt et al., 1995; Arrasate et al., 2000), a localization that could favor its further secretion. Also, tau is found present at various cell protrusions like dendritic spines (Ittner et al., 2010), growth cones (Dotti et al., 1987), axonal grains (Dennissen et al., 2016) and presynaptic compartments (Zhou et al., 2017). In the postsynaptic compartment, tau binds to presynaptic vesicles through the transmembrane vesicle protein synaptogyrin-3, as found in the brain of AD patients (McInnes et al., 2018). A reduction of synaptogyrin-3 prevents the association of presynaptic tau with vesicles and may facilitate neurotransmitter release (McInnes et al., 2018).

In contrast, an unconventional secretory pathway is the one involving protein secretion through extracellular vesicles such as exosomes and microvesicles. This mechanism has been proposed to decrease the levels of some intracellular proteins (Simons and Raposo, 2009). Wang et al. (2017) have demonstrated that tau may be released via exosomes by neurons or cultured cells and the release of exosomes is enhanced by neuronal activity. Furthermore, Asai et al. (2015) have suggested that microglia may phagocytize tau-containing neurons and would secrete tau in exosomes, in order to facilitate its propagation to neurons. Recently, Katsinelos et al. (2018) have characterized a mechanism in which hyperphosphorylated tau is secreted through direct translocation across the plasma membrane. PI(4,5)P_2_ and proteoglycans are involved in this secretion process.

Additionally, the involvement of larger extracellular vesicles, ectosomes, has been proposed (Dujardin et al., 2014).

**FIGURE 1:**
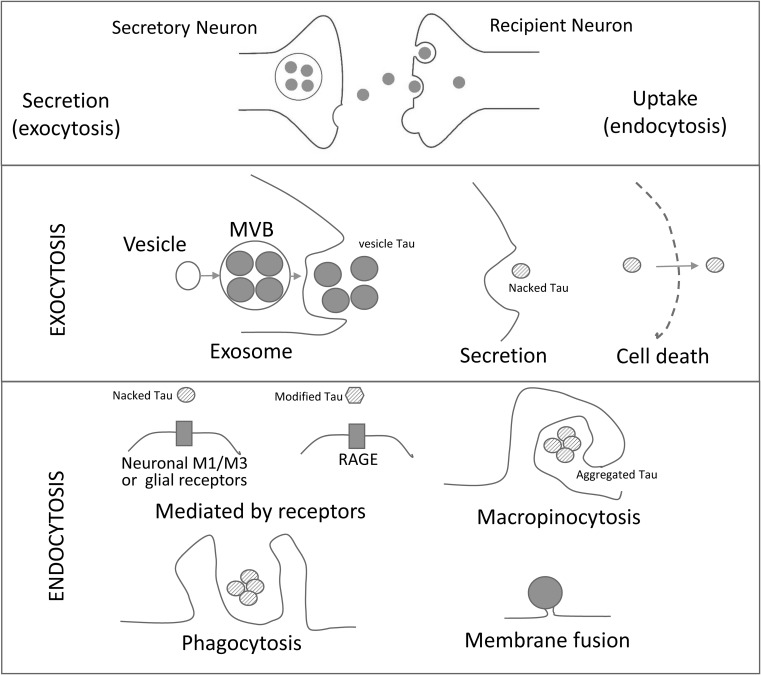
Schematic Representation of Cell-to-Cell Transmission of Tau Pathology. Tau seeds are released from neurons by different exocytosic mechanisms (exosome, secretion and neuronal death). The released tau is taken up by neurons or glial cells by a variety of mechanisms (mediated by receptors, micropinocytosis, phagocytosis and/or membrane fusion of exosomes. RAGE: receptor for advanced glycation endproducts. M1/M3: muscarinic receptors.

Multivesicular bodies (MVBs; late endocytic compartments) can fuse with the plasma membrane to release intraluminal vesicles into the extracellular medium, and once secreted, these vesicles are called the exosomes (Fuster-Matanzo et al., 2015) (Figure 1). In contrast, microvesicles arise by outward budding of the plasma membrane (Simons and Raposo, 2009; Gangalum et al., 2011).

## Exosomes and Interactions With the Cell Surface of Target Cells

Exosomes are defined as signaling organelles involved in health or in disease signaling pathways (Corrado et al., 2013). Measuring between 30 and 150 nm, these extracellular vesicles were first described almost 40 years ago (Trams et al., 1981). They are composed by a membrane that contains proteins like tetraspanins, flotillin and cell-specific receptors, and lipid rafts containing cholesterol, sphingomyelin and ceramide (DeLeo and Ikezu, 2018). The vesicle itself holds different types of proteins, nucleic acids (like miRNAs) or protein nucleic acid complexes depending of the cell types from which they are released (Corrado et al., 2013). Also, exosomes can also be found in several body fluids like plasma, saliva, urine, cerebrospinal fluid, amniotic fluid, colostrum, breast milk, synovial fluid, semen and pleural ascites (Corrado et al., 2013; McKelvey et al., 2015). For this reason, exosomes have been used as biomarkers of different diseases. Recently, a precipitation/immunoaffinity system has been developed to isolate neuron-derived as well as astrocyte-derived exosomes in the blood of Alzheimer’s disease patients. Results from these studies suggest that neuronal exosomes from blood plasma and that measurement of certain forms of tau in neuronal exosomes can be used as a diagnostic and prognostic biomarker to the disease (Goetzl et al., 2018; Guix et al., 2018).

The membranes of some types of exosomes contain proteins with the capacity to interact with the plasma membrane proteins of their target cells. For instance, B-lymphocytes exosomes bear integrin that are capable to interact with fibroblasts (Clayton et al., 2004). However, less specific links, mainly through the extracellular matrix (Corrado et al., 2013), may contribute to the interaction with target cells. In general, several mechanisms have been put forward to explain the interaction of exosomes with target cells that results in cell membrane fusion, phagocytosis, macropinocytosis, and receptor-mediated endocytosis (McKelvey et al., 2015) (Figure 1).

## Neuron-Derived Exosomes and Neurodegeneration

Neuron-derived exosomes containing specific proteins related to neurological disorders can be released from the neurons affected. In the case of AD, the presence of exosomes containing tau or amyloid-β peptide has been reported. Exosomes can transport the amyloid-β peptide or fragments of its precursor protein (APP; Rajendran et al., 2006; Sharples et al., 2008; Perez-Gonzalez et al., 2012; Sardar Sinha et al., 2018). Furthermore, aggregated tau has been reported in brain exosomes of mouse models of tauopathy (Baker et al., 2016; Polanco et al., 2016). In these animal models, neuronal exosomes containing human mutated tau are toxic to the recipient neurons *in vivo* (Winston et al., 2018). Also, tau exosomes could be used as biomarkers not only for AD but also Down syndrome and Parkinson’s disease (Shi et al., 2016; Hamlett et al., 2018). With respect to phosphorylation, tau secreted by exosomes is phosphorylated at some AD epitopes (Hampel et al., 2004; Saman et al., 2012).

Indeed, it has been suggested that the tau efflux from the Central Nervous System via exosomes is increased in Parkinson’s disease but not in AD (Shi et al., 2016). However, exosomes containing tau protein have been found in human biofluids in AD patients (Fiandaca et al., 2015; Guix et al., 2018) and the release and *trans*-synaptic transmission of tau via exosomes has been also described (Wang et al., 2017).

## Tau Transmission From Cell to Cell

The exact mechanism of tau release is unclear, and some studies have demonstrated that both vesicle-bound and soluble free extracellular populations of tau exist (Saman et al., 2012; Kanmert et al., 2015; Wang et al., 2017). Furthermore, neuron death results in the release of tau into the extracellular space. Soluble-free tau protein can interact with M1/M3 muscarinic receptors which may be present, not only in neurons but also in some glia (Santello et al., 2019). The reception of tau by these receptors may lead to an increase in intracellular calcium (Gomez-Ramos et al., 2008; Diaz-Hernandez et al., 2010). On the other hand, extracellular tau reacts with fractalkine receptors in microglia (Bolos et al., 2017; Perea et al., 2018) and this interaction may contribute to tau propagation (Asai et al., 2015).

Tau propagation implicates its cellular uptake by surroundings cells. Clathrin-mediated endocytosis, micropinocytosis, or direct membrane fusion have been proposed as possible mechanisms of tau uptake (Christianson and Belting, 2014; Calafate et al., 2016; McInnes et al., 2018).

The discovery of tau spreading has prompted several researchers to focus on the development of tau antibodies as immunotherapies to block the cell-to-cell transmission of pathological tau (Boutajangout et al., 2011; Chai et al., 2011; Yanamandra et al., 2013; Dai et al., 2018).

## Future

Further research should address exosomes containing tau. These vesicles contain proteins and nucleic acids and sometimes complexes of both. Tau is a nucleic acid-binding protein (Villasante et al., 1981; Sultan et al., 2011) and, inside the exosome, it can be bound to a specific nucleic acid. This binding could result in conformational changes in the tau molecule, which would lead to diverse tau-prion strains (Holmes et al., 2013; Sharma et al., 2018). In summary, a better characterization of tau isoforms present in exosomes would help us to understand the mechanism of tau propagation.

## Author Contributions

All authors listed have made a substantial, direct and intellectual contribution to the work, and approved it for publication.

## Conflict of Interest Statement

The authors declare that the research was conducted in the absence of any commercial or financial relationships that could be construed as a potential conflict of interest.
